# Establishment of a QuEChERS-FaPEx Rapid Analytical Method for *N*-Nitrosamines in Meat Products

**DOI:** 10.3390/molecules31010032

**Published:** 2025-12-22

**Authors:** Chun-Han Su, Peng-Wang Tan, Tsai-Hua Kao

**Affiliations:** 1 Department of Food Science, Fu Jen Catholic University, New Taipei City 242, Taiwan; 154286@mail.fju.edu.tw (C.-H.S.);; 2 Ph.D. Program in Nutrition and Food Science, College of Human Ecology, Fu Jen Catholic University, New Taipei City 242, Taiwan

**Keywords:** *N*-nitrosamines, QuEChERS-FaPEx, meats, LC-MS/MS

## Abstract

This study aimed to establish a fast and efficient method for the determination of *N*-nitrosamines (NAs) in meat products by integrating two sample preparation techniques—QuEChERS (Quick, Easy, Cheap, Effective, Rugged, and Safe) and FaPEx (Fast Pesticide Extraction)—with liquid chromatography–tandem mass spectrometry (LC–MS/MS). Chromatographic separation was performed on a Poroshell 120 Phenyl Hexyl column using a gradient elution of acetonitrile and 0.01% formic acid at a flow rate of 0.3 mL/min and a column temperature of 25 °C. Under these conditions, nine NAs and one internal standard were completely separated within 11 min with selective reaction monitoring mode (SRM) for detection. Samples were first extracted with QuEChERS powder using acetonitrile containing 0.1% formic acid, followed by purification with a FaPEx-Chl cartridge. This combined approach demonstrated superior performance compared with traditional solvent extraction or QuEChERS extraction alone. The recoveries of the developed method ranged from 76% to 111% and 52% to 103% at spiking levels of 50 ng/g and 20 ng/g, respectively. The limits of detection (LOD) and quantification (LOQ) were 0.002–0.3 ng/g and 0.006–1.00 ng/g, respectively. The inter-day and intra-day precisions (RSD%) ranged from 2.7% to 17% and 2.9% to 17%, respectively. These results indicate that the proposed method is among the most time-efficient and effective analytical approaches currently available for the determination of NAs in meat products.

## 1. Introduction

*N*-nitrosamines (NAs) are a class of compounds composed of carbon, hydrogen, oxygen, and nitrogen atoms, characterized by the presence of a nitroso group. According to the International Agency for Research on Cancer, *N*-nitrosodimethylamine (NDMA) and *N*-nitrosodiethylamine (NDEA) are classified as Group 2A carcinogens, while *N*-nitrosomethylethylamine (NMEA), *N*-nitrosopiperidine (NPIP), *N*-nitrosopyrrolidine (NPYP), *N*-nitrosodibutylamine (NDBA), *N*-nitrosodipropylamine (NDPA), and *N*-nitrosomorpholine (NMO) fall into Group 2B. *N*-nitrosodiphenylamine (NDPHA), by contrast, is categorized as Group 3. Given their toxicological relevance, the presence of NAs in food products represents a significant concern for food safety and public health.

NAs are primarily formed through reactions between nitrite and amine precursors. Under acidic conditions, nitrite is protonated to form nitroxylic acid (HNO_2_), which further converts to dinitrogen trioxide (N_2_O_3_). This reactive intermediate readily interacts with primary, secondary, or tertiary amines, resulting in the formation of various NAs [1,2]. Consequently, both fermented vegetables and nitrite-containing meat products are potential sources of NAs formation, with the latter generally exhibiting higher levels. The concentrations and profiles of NAs in meat products are influenced by multiple factors, including cooking method, temperature, duration, moisture content, fat composition, and residual nitrite levels [3]. For example, Chih et al. (2025) [4] quantified NDMA, NPYR, NDEA, NPIP, and NDBA in meat products subjected to different cooking conditions and reported that stir-fried cured meat stored for seven days exhibited the highest total NAs concentration (5.58–609 μg/kg), primarily comprising NDMA (<LOQ–595 μg/kg) and NPYR (<LOQ–27.7 μg/kg). Stored smoked chicken showed the next highest total NAs levels (5.25–142 μg/kg), again dominated by NDMA (3.06–140 μg/kg). Other products—such as ham, bacon, sausage, and salted fish—also contained detectable NAs, although at substantially lower total contents (0.25–16.7 μg/kg). Despite the relatively high NDMA concentrations observed in some meat samples, the literature also indicates that NDMA was not detected in the majority (114) of analyzed samples. In a separate study, Ozbay and Sireli (2021) [5] reported total volatile NAs concentrations of 7.86–29.11 μg/kg in salami. Beyond cured meat products, dried aquatic products have also been identified as NAs sources, with Huang et al. (2023) reporting an average NDMA content of 5.84 μg/kg [6].

Despite their toxicological significance, regulatory limits for NAs in food remain limited. The United Kingdom mandates that total volatile NAs in cured meats must not exceed 10 μg/kg, while China stipulates maximum NDMA levels of 4 μg/kg in fish and 7 μg/kg in fish products [7]. Most other nations regulate nitrite residues rather than NAs themselves. However, residual nitrite does not reliably predict NAs formation, emphasizing the need for analytical methods capable of directly determining NAs with high sensitivity and efficiency.

Conventional extraction techniques for NAs—including distillation, liquid–liquid extraction (LLE) [8], and solid-phase extraction (SPE) [9]—are often presented significant drawbacks. For example, distillation provides good recovery but is time-consuming, energy-intensive, and inefficient, particularly for non-volatile NAs. LLE is simpler but generates large volumes of waste and is laborious, while SPE reduces solvent consumption but remains complex and time-consuming. Therefore, if rapid sample preparation techniques, such as QuEChERS (Quick, Easy, Cheap, Effective, Rugged, and Safe) and Fast Pesticide Extraction (FaPEx), can be developed for the analysis of NAs, the shortcomings of the above methods may be improved.

Traditional QuEChERS consists of two processes: a dispersive extraction step and a dispersive solid-phase extraction (d-SPE) cleanup step. FaPEx involves solvent extraction of the analyte followed by direct passage of the crude extract through a cartridge packed with sorbents, which selectively adsorb impurities and thereby achieve sample cleanup. Since only a single purification step is involved, FaPEx is typically coupled with mass spectrometric analysis to minimize potential interferences.

Although its principle is similar to that of QuEChERS, the two approaches present distinct advantages and limitations. Unlike QuEChERS, FaPEx does not utilize extraction for salting-out, which may reduce analyte recovery. However, FaPEx employs packed sorbents, eliminating the centrifugation steps required by the dispersive cleanup in QuEChERS, making the procedure simpler and more time-efficient. Moreover, FaPEx can be tailored by modifying the sorbent composition within the cartridge to develop different kits (e.g., FaPEx-VAg, FaPEx-HP, and FaPEx-NM) for specific analytes such as veterinary drugs or samples with diverse chemical properties.

Fewer than ten publications have employed FaPEx technique, and exclusively for pesticide determination in agricultural products [10,11,12,13,14], environmental contaminants such as fipronil in soil and eggs [15], and the analysis of benzophenone, a chemical sensitizer, in breakfast cereals [16]. The above also shows that FaPEx applications have continuous expansion potential.

To date, only a limited number of studies have applied the QuEChERS approach for the extraction of NAs, with no applications reported for meat products. Moreover, FaPEx has not yet been utilized in the analysis of NAs. Therefore, this study aims to develop a rapid method combining QuEChERS and FaPEx for the extraction of NAs from food products. By integrating the strengths of these two fast-screening techniques, we expect to expand the analytical strategies available for NAs determination and enhance the efficiency of food contaminant monitoring.

## 2. Results and Discussion

### 2.1. Development of LC–MS/MS Method for Simultaneous Determination of Nine NAs

Three analytical columns—Hypersil Gold C18 (100 × 2.1 mm I.D., 1.9 μm), Poroshell 120 EC-C18 (100 × 4.6 mm I.D., 2.7 μm), and Poroshell 120 Phenyl Hexyl (150 × 2.1 mm I.D., 2.7 μm)—were evaluated using various mobile phases and modifiers to identify optimal chromatographic conditions. The best separation performance was obtained using the Poroshell 120 Phenyl Hexyl column (Agilent Technologies, Polo Alto, CA, USA). The mobile phase system consisted of (A) acetonitrile and (B) 0.1% formic acid in water, with the following gradient: 95% B at 0 min, 90% B at 3 min, 80% B at 6 min, 10% B at 10 min, held for 3 min, then returned to the initial condition (95% B) at 14 min and equilibrated for 2 min. The flow rate was set at 0.3 mL/min, and the column temperature was maintained at 25 °C. Under these conditions, all nine NAs and one internal standard were separated within 11 min. Table 1 summarizes the precursor and product ions used for the qualitative and quantitative MS/MS analysis of each n-nitrosamine.

Currently, the analysis of NAs is still predominantly performed using GC–MS, mainly because several NAs exhibit volatile characteristics. Nevertheless, an increasing number of LC–MS/MS methods have been developed. Compared with recent studies, Li et al. (2019) [17] employed an Eclipse Plus C18 column (100 × 2.1 mm I.D., 3.5 μm) with methanol and 0.1% formic acid in aqueous solution as the mobile phases, enabling the determination of five NAs. Huang et al. (2023) [6] utilized an HSS T3 column (100 × 2.1 mm I.D., 1.8 μm) with methanol and 0.05% formic acid in aqueous solution as the mobile phases, allowing the analysis of four NAs. Niklas et al. (2022) [18] applied a Poroshell Phenyl-Hexyl column (150 × 2.1 mm I.D., 3 μm) with methanol and 0.1% formic acid in aqueous solution as the mobile phases and successfully determined seven NAs. Chen et al. (2022) [19] used a Shim-pack GIST C18 column (100 × 2.1 mm I.D., 2 μm) with methanol and 5 mM ammonium acetate aqueous solution containing 0.1% formic acid as the mobile phases, achieving the simultaneous determination of seven NAs. The column and mobile phase used in our study were the same as those in Niklas et al. (2022) [18], but a larger number of NAs were separated in our study.

Compared with the aforementioned studies, the method developed in the present study allows for the determination of a larger number of NAs. To date, the LC–MS/MS method reported by Giménez–Campillo et al. (2025) [20] enables the separation of the highest number of NAs, employing an ACQUITY UPLC HSS T3 column (100 × 2.1 mm I.D., 1.8 μm) with 5 mM ammonium acetate aqueous solution and 0.1% formic acid aqueous solution as the mobile phases, and allowing the analysis of up to 13 NAs. However, this method was operated in APCI mode, which differs from the ESI mode used in the present study. Although the number of NAs analyzed in this study is lower than that reported research, the target compounds selected herein comprehensively cover the NAs most frequently detected in meat products and of greater toxicological and regulatory concern.

### 2.2. Optimization of Condition for QuEChERS Extraction Plus FaPEx Clean Up

#### 2.2.1. Evaluation of FaPEx Cartridge Types

Five FaPEx cartridges—FaPEx-Gen, FaPEx-Cer, FaPEx-VAG, FaPEx-Tea, and FaPEx-Chl—originally designed for pesticide residue analysis in fruits and vegetables, cereals, β-agonist matrices, tea, and high-chlorophyll samples, respectively, were assessed for their suitability in NAs extraction.

**Figure 1:**
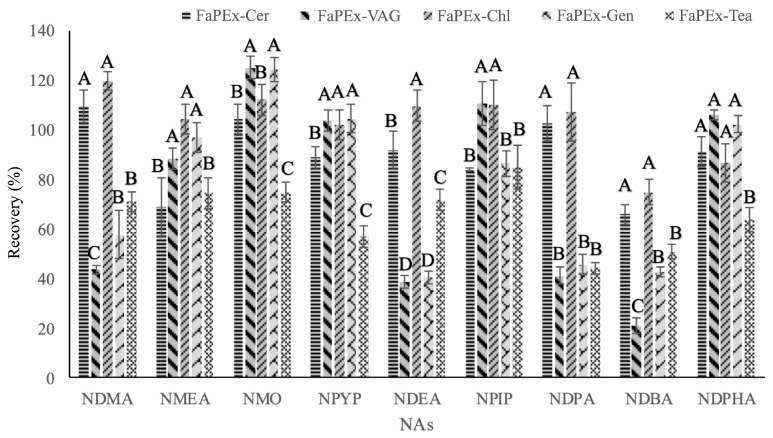
Effect of different FaPEx cartridges on the recovery of 9 NAs from pork belly. Data with different capital letters in the same NAs are significantly different at *p* < 0.05. The full names corresponding to the abbreviations of NAs are provided in Table 1.

As shown in Figure 1, after QuEChERS extraction with acetonitrile followed cleanup by different FaPEx cartridges, recoveries of nine NAs varied considerably among the cartridges. Overall, FaPEx-Chl yielded the highest recoveries (76–122%), while FaPEx-VAG and FaPEx-Tea produced the lowest (21–125% and 48–85%, respectively).

No prior studies have reported the application of FaPEx cartridges for NAs purification, and the underlying mechanism remains unclear. However, according to Zeng et al. [21], when using the QuEChERS method to extract NAs from soy sauce, increasing the amount of graphite carbon black (GCB) in the cleanup sorbent from 50 to 150 mg first improved and then decreased the recoveries of NDBA, NPIP, and NPYP. Similarly, NDPA, NDEA, and NDMA showed decreased recoveries at higher GCB levels. In the present study, FaPEx-Chl, which exhibited the best performance, contains an intermediate GCB amount between FaPEx-Tea and FaPEx-Gen (the poorest performers). This suggests that excessive or insufficient GCB negatively affects NAs recovery, while a moderate amount facilitates effective purification by balancing matrix removal and minimizing NAs adsorption losses.

#### 2.2.2. Evaluation of Extraction Solvents

**Figure 2:**
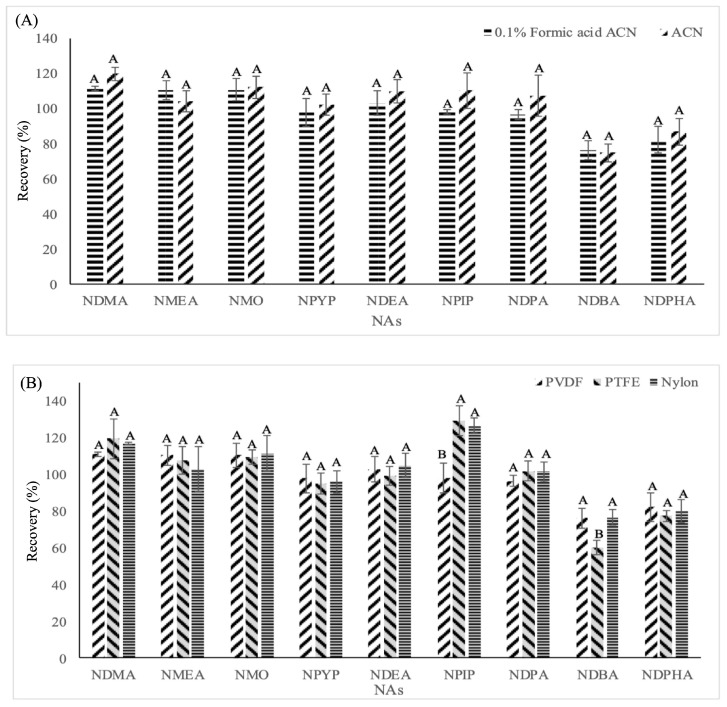

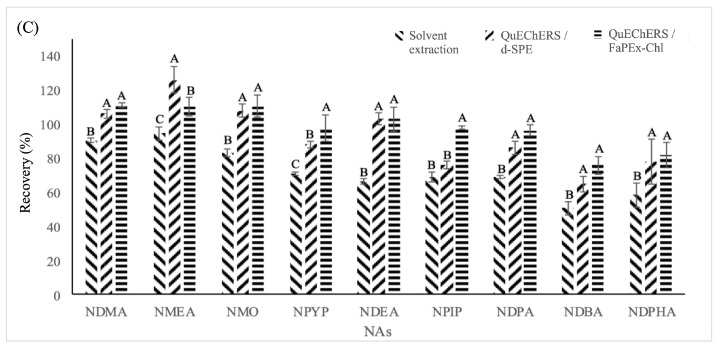
The recoveries of the nine NAs in pork belly extracted by QuEChERS/FaPEx-Chl under different extraction conditions. (**A**) different solvents, (**B**) different syringe filters, and (**C**) different extraction methods. Data with different capital letters in the same NAs are significantly different at *p* < 0.05. The full names corresponding to the abbreviations of NAs are provided in Table 1.

Since FaPEx-Chl yielded the best performance, it was selected to evaluate the effect of different QuEChERS extraction solvents—acetonitrile and 0.1% formic acid in acetonitrile. As shown in Figure 2A, the recovery rates using acetonitrile and acetonitrile containing 0.1% formic acid showed negligible differences. However, for certain NAs, such as NDMA and NPIP, the use of acetonitrile with 0.1% formic acid resulted in better repeatability (indicated by a smaller standard deviation). Furthermore, acidified ACN also reduced matrix-induced ion suppression in LC–MS/MS, resulting in more stable quantitation. Although the average recovery is slightly lower, its accuracy and reproducibility are more stable, making it suitable as a final method.

#### 2.2.3. Evaluation of Filter Membrane Materials

Given that NAs include both polar and non-polar compounds, adsorption by filter membranes could affect recoveries. The effects of different filter types—PVDF, Nylon (polar), and PTFE (non-polar)—were evaluated. As shown in Figure 2B, filter type had little influence on most NAs; however, for some compounds, such as NDBA, PVDF, and Nylon, yielded significantly higher recoveries than PTFE. Therefore, PVDF was selected as the optimal membrane material for subsequent analyses.

### 2.3. Comparison Among Solvent Extraction, QuEChERS Extraction Plus d-SPE Clean Up (QuEChERS/d-SPE), and QuEChERS Extraction Plus FaPEx-Chl Clean Up (QuEChERS/FaPEx-Chl)

Figure 2C compares the recoveries obtained using three extraction approaches. For all NAs, solvent extraction exhibited the lowest recoveries. The QuEChERS/d-SPE and the QuEChERS/FaPEx-Chl gave comparable recoveries for NDMA, NMO, NDEA, NDPA, NDBA, and NDPHA. However, recoveries of NPYP and NPIP were higher using the QuEChERS/FaPEx-Chl. Although NMEA showed a slightly higher recovery with QuEChERS/d-SPE alone, the QuEChERS/FaPEx-Chl method achieved values closer to 100% with lower variability. Overall, the QuEChERS/FaPEx-Chl approach provided the most consistent and highest recoveries.

The solvent extraction recoveries in this study (51–95%) were comparable to those reported by Herrmann et al. (2014) [22] (57–86% at 30 ng/g spiking), though higher recoveries for NMEA and NDMA were achieved here. Although QuEChERS-based extraction of NAs from meat has not been reported previously, similar applications in other matrices have yielded satisfactory results—for example, soy sauce (81–112%) [21], Chinese salted fish (87–123%) [23], and Sichuan salted vegetables (88–105%) [24]. The present study demonstrated recoveries of 65–126% for pork, indicating the feasibility of applying QuEChERS/FaPEx-Chl for NAs determination in meat products.

In addition, the integration of QuEChERS with FaPEx significantly reduces the number of extraction and purification steps compared with conventional approaches such as distillation, liquid–liquid extraction, and SPE, which typically require multiple shaking, phase transfer, concentration, or cartridge-conditioning procedures. Moreover, the one-step purification of the FaPEx cartridge eliminates centrifugation and multi-step cleanup, thereby markedly shortening the total sample pretreatment time. Based on the comparative results shown in Figure 2C, the entire workflow reduces sample preparation time, and chromatographic separation of nine NAs is completed within 11 min. In summary, FaPEx-Chl cleanup is simpler and faster than conventional QuEChERS procedures.

### 2.4. Method Validation

#### 2.4.1. Limits of Detection (LOD) and Quantitation (LOQ)

Table 2 summarizes the LODs and LOQs for nine NAs obtained with the developed LC–MS/MS method. Compared with previous LC–MS/MS studies, the present method achieved lower limits: Qian et al. [25] reported LODs of 0.04–2.67 ng/g and LOQs of 0.12–8.78 ng/g for drinking water, while Herrmann et al. [22] reported LODs of 0.05–3.30 ng/g and LOQs of 0.10–6.70 ng/g in processed meats using APCI mode.

Among all NAs, NDPHA exhibited the lowest LOD, likely due to its nonvolatile nature, resulting in less analyte loss within the LC–MS/MS system. Similar trends were reported by Qian et al. [25]. Moreover, the LOD for NDPHA obtained here was even lower than that in previous studies, indicating superior sensitivity.

In contrast, GC–MS/MS studies have shown little effect of volatility on LOD. For example, Qiu et al. [23] reported identical LODs (0.01 ng/g) for NDPHA and volatile NAs such as NDMA, NMO, NMEA, and NDBA. Nonetheless, the LOD for NDPHA achieved in this study (0.002 ng/g) was markedly lower, demonstrating the higher analytical performance of the developed LC–MS/MS system.

For volatile NAs, LOQs ranged from 0.045 to 1.0 ng/g, which are higher than those reported by Qiu et al. [23] using QuEChERS–GC–MS/MS (0.03–0.33 ng/g). This indicates that GC remains advantageous for volatile NAs. Nevertheless, considering regulatory limits—e.g., <10 ng/g for total volatile NAs in cured meats (UK) and 4–7 ng/g for NDMA in fish products (China) [7]—the LOQs achieved here are well below these thresholds, confirming that the developed LC–MS/MS system provides sufficient sensitivity for regulatory monitoring.

#### 2.4.2. Precision

According to the “Guidelines for Validation of Food Chemical Analytical Methods” issued by the Taiwan Food and Drug Administration [26], when the target concentration range is 0.001–0.01 ppm, repeatability (intra-day variability) and intermediate precision (inter-day variability) should be below 30% and 32%, respectively. The developed QuEChERS/FaPEx-Chl method yielded intra-day RSDs of 2.9–17.1% and inter-day RSDs of 2.69–16.76% (Table 2), demonstrating satisfactory precision.

#### 2.4.3. Accuracy

According to the Taiwan FDA “Guidelines for Validation of Food Chemical Analytical Methods” [26], when analyte concentrations are ≤0.001 ppm, the acceptable recovery range is 50–125%. Using the developed method, recoveries for samples spiked with 50 ng/g and 20 ng/g of NAs ranged from 76% to 111% and 52% to 103%, respectively (Table 2), meeting the required validation criteria.

#### 2.4.4. Matrix Effect

Matrix effects refer to signal enhancement or suppression caused by co-eluting components in LC–MS/MS analysis. Although no official regulatory limit exists, effects within ±20% are generally considered minimal [22]. In this study, matrix effects for NAs extracted from pork using the QuEChERS/FaPEx-Chl method ranged from 2.7% to 73%. Only NDMA exhibited a matrix effect below 20%; the others exceeded this range. However, since internal standardization and matrix-matched calibration were applied, and all calibration curves exhibited excellent linearity (R^2^ = 0.995–0.999), quantification accuracy was not compromised, as further supported by the satisfactory recovery results.

### 2.5. Determination of NAs in Commercial Food Samples

The developed QuEChERS/FaPEx-Chl method was applied to analyze nitrite-containing commercial foods, including grilled bacon, grilled German sausage, raw sausage, Chinese preserved sausage, and canned luncheon meat. The results are presented in Table 3.

NAs were not detected in raw sausage and Chinese preserved sausage. In contrast, NDMA, NDBA, and NDPHA were found in grilled bacon; NDMA and NDPHA in grilled German sausage; and NDBA and NDPHA in canned luncheon meat. These findings suggest that although nitrite was present in all samples, unheated products such as raw sausages did not form detectable NAs. Heat processing (e.g., grilling or canning); however, promoted NA formation—particularly NDMA, NDBA, and NDPHA, with NDPHA showing the highest levels.

Previous studies have similarly reported that proteins and lipids in meat can form amine precursors during heating, fermentation, smoking, and curing, which subsequently react with nitrite or nitrogen oxides to produce NAs [1,27]. Mirzazadeh et al. [28] also demonstrated that grilling produced the highest NA levels, followed by frying, whereas microwaving generated the least. Higher cooking temperatures significantly increased the NA formation.

Numerous studies have demonstrated that uncured raw meat generally contains low levels of NAs or even non-detectable concentrations. This is primarily because nitrite has not yet been added, resulting in relatively low levels of secondary amines, and because the meat has not undergone high-temperature processing, fermentation, or drying, all of which can promote nitration [29,30]. In contrast, cured meat products typically contain significantly higher levels of NAs due to the intentional addition of nitrite and the increased availability of secondary amines arising from protein degradation. Furthermore, storage and ripening processes facilitate nitration, thereby contributing to elevated NAs formation compared with raw meat [18].

When cured meat products are further subjected to thermal processing, such as cooking, frying, or smoking, NAs concentrations may increase even further. This enhancement is attributed to the acceleration of lipid oxidation at elevated temperatures, leading to increased free radical generation that promotes nitration, as well as moisture loss during heating, which results in a concentration effect [30,31]. The above mechanisms can help explain the observed increase in NAs levels in cured meat following high-temperature processing.

## 3. Materials and Methods

### 3.1. Materials

Meat products were purchased from local convenience stores. Prior to analysis, samples were homogenized using a grinder, placed in amber sample vials, and stored at −20 °C.

### 3.2. Chemicals and Reagents

Nine NAs standards, including *N*-nitrosodiethylamine (NDEA), *N*-nitrosomethylethylamine (NMEA), *N*-nitrosopiperidine (NPIP), *N*-nitrosopyrrolidine (NPYP), *N*-nitrosodimethylamine (NDMA), *N*-nitrosodibutylamine (NDBA), *N*-nitrosodiphenylamine (NDPHA), *N*-nitrosodipropylamine (NDPA) and *N*-nitrosomorpholine (NMO) as we as internal standard *N*-nitrosopyrrolidine-d8 (NPYP-d8) were from Toronto Research Chemicals Co., Ltd. (Downsview, ON, Canada). HPLC-grade solvents were purchased from Merck (Darmstadt, Germany). Deionized water was produced using the arium^®^ pro water purification system manufactured by Sartorius (Goettingen, Germany). The QuEChERS set included extraction powder (contained 4 g of anhydrous magnesium sulfate and 1 g of anhydrous sodium acetate), purification powder (contained 300 mg of primary and secondary amine, 900 mg of MgSO_4_, and 300 mg of C18EC), homogenizers, and centrifuge tubes was purchased from Uni-Onward Corp (Taipei, Taiwan). FaPEx cartridge included FaPEx-Gen, FaPEx-Cer, FaPEx-VAG, FaPEx-Tea, and FaPEx-Chl were from GETECH Co. (Kaohsiung, Taiwan).

### 3.3. Instrumentation

Dionex UltiMate 3000 Open Sampler XRS System UPLC, and TSQ Quantiva triple quadrupole tandem mass spectrometer were from Thermo Fisher Scientific (San Jose, CA, USA). Poroshell 120 Phenyl Hexyl column (150 × 2.1 mm I.D., 2.7 μm) as well as Poroshell 120 Phe-Hex guard Column (5 × 2.1 mm I.D., 2.7 μm) were from Agilent Technologies (Polo Alto, CA, USA).

### 3.4. Analysis of NAs by LC-MS/MS

A triple-quadrupole tandem mass spectrometer with positive electrospray ionization (ESI+) and SRM was used for compound detection at a spray voltage of 3500 V, collision gas of 1.5 arbitrary units, sweep gas flow rate of 0 aritrary units, sheath gas flow rate of 38 arbitrary units, auxiliary gas flow rate of 12 arbitrary units, ion transfer tube temperature of 329 °C, and vaporizer temperature of 279 °C. The various NAs in samples were detected according to their elution order and specific *m*/*z* as described in Table 1, which shows the operation parameters such as precursor ion as well as product ion (quantitation ion and confirmation ion) along with their corresponding collision energy used for differentiating each furan and its derivatives.

### 3.5. Evaluation of the Effectiveness of Different Methods for NAs Extraction

#### 3.5.1. Solvent Extraction

The extraction procedure was adapted from Hernnam et al. [22]. Two grams of blank samples (boiled pork belly slices) were weighed into 50 mL centrifuge tubes. One group was spiked with 100 μL of a 1 μg/mL mixed methanolic standard solution containing nine NAs, while the other group was unspiked. All samples were extracted with 7.5 mL of acetonitrile containing 0.1% formic acid by shaking for 10 min, followed by standing at −30 °C for 30 min. Samples were then centrifuged at 3800× *g* for 10 min at 0 °C, and the supernatant was collected. One milliliter of the supernatant was filtered through a 0.2 μm PVDF membrane. Then, 180 μL of the filtrate was mixed with 20 μL of 1 ppm NPYP-d8 (internal standard) and analyzed by LC–MS/MS to determine recovery efficiency.

#### 3.5.2. QuEChERS Extraction Plus d-SPE Cleanup (QuEChER/d-SPE)

Two grams of blank samples (boiled pork belly slices) were weighed into 50 mL centrifuge tubes. One group was spiked with 100 μL of a 1 μg/mL methanolic standard solution containing nine NAs, and the other was unspiked. Ceramic homogenization stones were added to each tube, followed by 10 mL of deionized water and 10 mL of acetonitrile. After shaking for 3 min, the samples were kept at −30 °C for 30 min. Subsequently, 5 g of QuEChERS extraction powder (1 g NaCl and 4 g anhydrous MgSO_4_) was added, vortexed vigorously for 30 s, and centrifuged at 3800× *g* for 10 min at 0 °C. The supernatant was collected, and 5 mL of it was mixed with a QuEChERS d-SPE cleanup powder (300 mg C18EC, 300 mg PSA, and 900 mg MgSO_4_), vortexed for 30 s, and centrifuged again under the same conditions. One milliliter of the final supernatant was filtered through a 0.2 μm PVDF membrane, and 180 μL of filtrate was mixed with 20 μL of 1 ppm NPYP-d8 (internal standard) for LC–MS/MS analysis. Extraction recovery was calculated to evaluate the efficiency of the procedure.

#### 3.5.3. QuEChERS Extraction Plus FaPEx-Chl Cleanup (QuEChERS/FaPEx-Chl)

##### Evaluation of FaPEx Cartridge

Five milliliters of the QuEChERS extraction supernatant prepared in Section 3.5.2 were loaded into five different FaPEx kits (FaPEx-Gen, FaPEx-Cer, FaPEx-VAG, FaPEx-Tea, and FaPEx-Chl) for purification. The filtrate was collected and further filtered through a 0.2 μm PVDF membrane. A 180 μL aliquot of the filtrate was mixed with 20 μL of 1 ppm NPYP-d8 (internal standard) and analyzed by LC–MS/MS to determine recovery efficiency.

##### Evaluation of QuEChERS Extraction Solvents

Following the procedure described in Section 3.5.2, 10 mL of acetonitrile or acetonitrile containing 0.1% formic acid was used as the QuEChERS extraction solvent. After vigorous shaking for 3 min and standing at −30 °C for 30 min, 5 g of QuEChERS extraction powder (1 g NaCl and 4 g anhydrous MgSO_4_) was added, vortexed for 30 s, and centrifuged at 3800× *g* for 10 min at 0 °C. Five milliliters of the resulting supernatant were used for subsequent FaPEx purification tests.

##### Evaluation of Syringe Filter Membrane Types

The effects of polar (PVDF and Nylon) and non-polar (PTFE) syringe filters on NA recoveries were evaluated. After extraction using the optimal solvent, the extracts were filtered using PVDF (13 mm × 0.22 μm), Nylon (13 mm × 0.22 μm), or PTFE (13 mm × 0.22 μm) membranes. Recoveries were compared to assess their influence on analyte loss.

### 3.6. Validation of Analytical Performance

#### 3.6.1. Limits of Detection (LOD) and Quantitation (LOQ)

A series of low-concentration matrix-matched mixed standard solutions was analyzed by MS/MS. The detection limit (LOD) was defined as a signal-to-noise ratio (S/N) ≥ 3 for the quantifier ion, while the quantitation limit (LOQ) corresponded to S/N ≥ 10.

#### 3.6.2. Recovery Tests

NAs’ standard solutions were spiked into boiled pork belly (blank samples) at two concentrations (20 ng/g and 50 ng/g). Samples were processed using the optimized extraction and purification procedures and analyzed by LC–MS/MS. Recovery (%) was calculated as follows:

Recovery (%) = [(spiked amount + original amount) − (original amount)]/spiked amount.

#### 3.6.3. Precision (Repeatability and Intermediate Precision)

For intra-day precision, a single extract was analyzed five times by the same operator on the same day, and the relative standard deviation (RSD) was calculated as repeatability. For inter-day precision, the same extract was analyzed five times per day on three different days (n = 15), and the overall RSD was reported as intermediate precision.

#### 3.6.4. Matrix Effect

Ten NAs standard solutions were prepared both in methanol (solvent calibration curve, SCC) and in the sample extract (matrix-matched calibration curve, MCC) at concentrations of 1, 5, 10, 25, 50, and 100 ng/mL. The peak areas (*A*) obtained from UPLC–MS/MS analysis were compared to calculate matrix effects as follows:

Matrix effect (%) = (*A*_matrix_/*A*_solvent_ − 1) × 100%

### 3.7. Quantification of NAs

Matrix-matched calibration was employed for the quantification of NAs in this study. Methanolic standard solutions containing ten NAs at concentrations of 1, 5, 10, 25, 50, 100, and 200 ng/mL, along with an internal standard (NPYP-d8) at 20 ng/mL, were prepared. An aliquot of 130 μL of the extract from pork belly by 0.1% formic acid in ACN, previously filtered through a PVDF membrane filter, was transferred into a vial, followed by the addition of 20 μL of a 1 ppm internal standard solution (NPYP-d8). Appropriate volumes of the NAs standard solutions were then added to achieve the desired calibration levels, and methanol was added to bring the final volume to 200 μL. The prepared solutions were subsequently analyzed using LC–MS/MS. The calibration curve was constructed by plotting the peak area ratio of analyte to internal standard (*A_S_*/*A_i_*) against the corresponding concentration ratio (*C_S_*/*C_i_*). Quantification of NAs in samples was performed using the regression equation obtained from the calibration curve. The following formula was subsequently applied for calculation:

Concentration of NAs (ng/g) = {[(*A*/RRF)/*A_i_*] × *C_i_*}/weight of sample (g)

where *A* is the peak area for NAs; RRF is the relative response factor, equal to (*A*/*A_i_*)/(*C*/*C_i_*); *A_i_* is the peak area for the internal standard, and *C_i_* is the concentration of the internal standard.

### 3.8. Statistical Analysis

The LC–MS/MS conditions, extraction methods, and NA contents in meat samples were performed in triplicate. Precision analysis was performed with five replicates. Data were processed using Xcalibur software (version 4.3). Calibration curves, correlation coefficients, and recoveries were calculated in Microsoft Excel. Statistical analyses were conducted using SPSS 18. Variance was analyzed by ANOVA, and significant differences were determined using Scheffé’s test (α = 0.05).

## 4. Conclusions

By integrating QuEChERS extraction with FaPEx-Chl clean up, this study developed a rapid and efficient method for determining NAs in meat products. The combined approach offers higher recoveries and greater convenience than QuEChERS alone and was successfully applied to real commercial samples. Furthermore, the results indicate that nitrite-containing meat products readily form NAs during heating; thus, reducing cooking temperature and duration is recommended as a practical measure to mitigate NAs formation in meat.

## Figures and Tables

**Table 1:** Operation parameters of 9 NAs standards and one internal standard in selective reaction monitoring (SRM) mode by UPLC-MS/MS.

NAs	Precursorion (*m*/*z*)	Quantitation		Confirmation	
		Production (*m*/*z*)	Collision Energy (V)	Production (*m*/*z*)	Collision Energy (V)
*N*-nitrosodiethylamine (NDEA)	102	75	15	29	22
*N*-nitrosomethylethylamine (NMEA)	88	61	12	43	13
*N*-nitrosopiperidine (NPIP)	114	69	15	41	20
*N*-nitrosopyrrolidine (NPYP)	100	55	16	41	17
*N*-nitrosodimethylamine (NDMA)	74	43	10	58	14
*N*-nitrosodibutylamine (NDBA)	158	103	10	57	14
*N*-nitrosodiphenylamine (NDPHA)	198	169	13	66	26
*N*-nitrosodipropylamine (NDPA)	130	89	11	43	13
*N*-nitrosomorpholine (NMO)	116	86	14	73	14
*N*-nitrosopyrrolidine-d8 (NPYP-d8)	106	62	10	46	15

**Table 2:** Limit of detection (LOD) ^1^, limit of quantitation (LOQ) ^2^, Matrix effect, recovery (%) ^3^, intra-day variability, and inter-day variability of 9 NAs detected by LC-MS/MS.

NAs	LOD (ng/g) ^1^	LOQ (ng/g) ^2^	Matrix Effect	Recovery (%) ^3^		Variability (RSD%) ^4^	
				50 ng/g	20 ng/g	Inter-Day	Intra-Day
NDMA	0.088	0.294	2.700	111 ± 1.15	52.1 ± 2.16	7.3	2.9
NMO	0.250	0.833	55.63	110 ± 5.31	78.1 ± 8.88	4.9	4.9
NPYP	0.060	0.200	49.32	110 ± 6.57	62.7 ± 2.27	5.4	5.4
NMEA	0.037	0.122	31.97	98.0 ± 7.92	103 ± 5.80	2.7	3.8
NDEA	0.100	0.333	27.24	103 ± 6.96	57.9 ± 0.78	6.0	3.2
NPIP	0.300	1.000	33.65	98.0 ± 1.18	65.1 ± 5.95	4.6	5.6
NDPA	0.188	0.625	62.19	96.0 ± 3.11	54.3 ± 2.47	17	4.2
NDBA	0.013	0.045	73.45	76.0 ± 5.19	65.8 ± 1.57	15	17
NDPHA	0.002	0.006	53.19	82.0 ± 7.63	54.3 ± 5.66	8.7	6.3

The full names corresponding to the abbreviations of NAs are provided in Table 1. ^1^ LOD (ng/g) based on S/N ≥ 3. ^2^ LOQ (ng/g) based on S/N ≥ 10. ^3^ Recovery (%) = (amount found—original amount)/amount spike × 100%. ^4^ RSD (%) = (SD/mean) × 100%.

**Table 3:** Contents of NAs (ng/g) in commercially available meat.

NAs	Grilled Bacon	Grilled German Sausage	Raw Sausage	Chinese Preserved Sausage	Pork Luncheon Meat
NDMA	6.42 ± 1.74	3.74 ± 0.12	N.D. ^1^	N.D.	N.D.
NMEA	N.D.	N.D.	N.D.	N.D.	N.D.
NMO	N.D.	N.D.	N.D.	N.D.	N.D.
NPYP	N.D.	N.D.	N.D.	N.D.	N.D.
NDEA	N.D.	N.D.	N.D.	N.D.	N.D.
NPIP	N.D.	N.D.	N.D.	N.D.	N.D.
NDPA	N.D.	N.D.	N.D.	N.D.	N.D.
NDBA	15.46 ± 0.61	N.D.	N.D.	N.D.	18.87 ± 0.12
NDPHA	31.1 ± 5.03	2.43 ± 0.49	N.D.	N.D.	48.55 ± 1.78

^1^ N.D: Not detected. The full names corresponding to the abbreviations of NAs are provided in Table 1.

## Data Availability

The original contributions presented in this study are included in the article. Further inquiries can be directed to the corresponding author(s).
